# Optimizing adherence to medication to improve outcomes in asthma

**DOI:** 10.1097/MCP.0000000000001166

**Published:** 2025-03-19

**Authors:** Pamela Rackow, Amelia Drennan, Hilary Pinnock, Alexandra L. Dima

**Affiliations:** a University of Stirling, Faculty of Natural Sciences, Psychology, Stirling; b Usher Institute, The University of Edinburgh, Edinburgh, UK; c Avedis Donabedian Research Institute (FAD) - Universitat Autònoma de Barcelona (UAB); d Avaluació de tecnologies sanitàries en atenció primària i salut mental (PRISMA), Institut de Recerca Sant Joan de Déu (IRSJD); e Consortium ‘Centro de Investigación Biomédica en Red’ Epidemiology and Public Health (CIBERESP), Spain

**Keywords:** asthma, behavioral interventions, medication adherence, self-management

## Abstract

**Purpose of review:**

Adherence to medication is essential for asthma control and reducing the risk of exacerbations. Research has accumulated in recent years on causes and consequences of adherence and effective interventions. This review highlights current advances in adherence research and their potential for clinical practice.

**Findings:**

Optimizing adherence to medication can be achieved through interventions that identify individual barriers and train the care team in offering tailored support. Digital technologies that facilitate remote monitoring, patient–provider communication and care coordination are increasingly being integrated into asthma care.

**Summary:**

Adherence determinants reported cover individual, social and health service-related factors. Age and attitudes toward adherence are crucial determinants. Patients’ and caregivers’ mental health is relevant for adherence and clinical outcomes, highlighting the importance of integrating this aspect into holistic asthma management. Single-site care arrangements are beneficial for adherence. Tailoring adherence interventions to individual needs, using brief questionnaires to assess barriers and recommending evidence-based strategies to address them, have been found useful and feasible across care settings. Digital technologies such as smart inhaler systems and telemedicine-enhanced care have been shown to be effective in randomized controlled trials, yet implementation research highlights challenges to sustaining support on the long-term.

## INTRODUCTION

Taking medication as prescribed is an essential component of asthma management and has been proven to contribute to improved symptom management and reduced risk of asthma exacerbations [1,2]. Nevertheless, patients continue to find it difficult to achieve optimal adherence, and healthcare professionals (HCP) often struggle with providing appropriate support [3]. Although the Global Initiative for Asthma (GINA) recommends reviewing inhaler technique and adherence to medications at every consultation as part of routine asthma assessment and management [1], following this recommendation is challenging in everyday practice. Supporting adherence effectively requires understanding what effect suboptimal adherence has on outcomes in different patient groups, what factors influence medication-taking behaviors at different levels, and what interventions are best suited to address these factors and can be implemented in specific clinical contexts. Moreover, as asthma is a variable condition, supported self-management can empower patients to adjust their preventer medication according to their asthma action plan.

Adherence to medications has been conceptualized as a complex group of behaviors and interactions [4,5] embedded in the broader process of asthma care. Research evidence is accumulating on the impact of these behaviors on a range of clinical outcomes, in particular severe exacerbations, hospitalizations, and deaths [6,7]. Preventing asthma deaths through appropriate medical management and education needs to be a priority for health systems [8]. Clinical teams need proven, evidence-based tools that they can easily integrate into routine patient-centered asthma management protocols [9]. Designing effective tools and interventions for specific patient groups and clinical contexts requires understanding the most relevant influencing factors (determinants) and selecting adherence support strategies capable of influencing these factors in the given context. Numerous determinants of medication adherence in asthma have been identified at patient level, HCP level, and healthcare system level [10,11]. Therefore, solutions for optimizing adherence need to support change simultaneously at patient level by supporting patients to understand their condition better and feel motivated to follow the prescribed regimen, and at the HCP and system levels by improving care processes and providing the necessary training and resources for guideline-recommended care. Research evidence supports this process by raising awareness on the value of optimizing adherence considering its impact on clinical outcomes, identifying and conceptualizing key modifiable determinants, and developing a range of solutions and testing their effectiveness and implementability in different clinical settings. This review highlights recent research on asthma medication adherence concerning these three key domains of research and reflects on their relevance for clinical practice.

A literature search was undertaken in December 2024 in PubMed using keywords related to ‘adherence’ and ‘asthma’. Selection criteria were language of publication (English), year of publication (2024), and type of study (peer-reviewed empirical study on adherence outcomes and/or determinants or development, evaluation, and/or implementation of adherence interventions in asthma). Study protocols, editorials, commentaries, opinion, and review articles were excluded. Three authors (P.R., A.D., A.L.D.) performed title and abstract screening, followed by full-text screening. Disagreements were resolved through discussion. Selected full-text articles were categorized into observational studies on adherence outcomes and/or adherence determinants, and evaluation or implementation science studies on adherence interventions, and summarized narratively. The literature search resulted in 221 references, of which 35 were selected for full-text review. Twenty-eight articles met inclusion criteria, of which 21 articles examined the impact of adherence on clinical outcomes and/or causes of suboptimal adherence in different patient groups, and 7 articles reported on the evaluation and/or implementation of adherence interventions. 

**Box 1:**
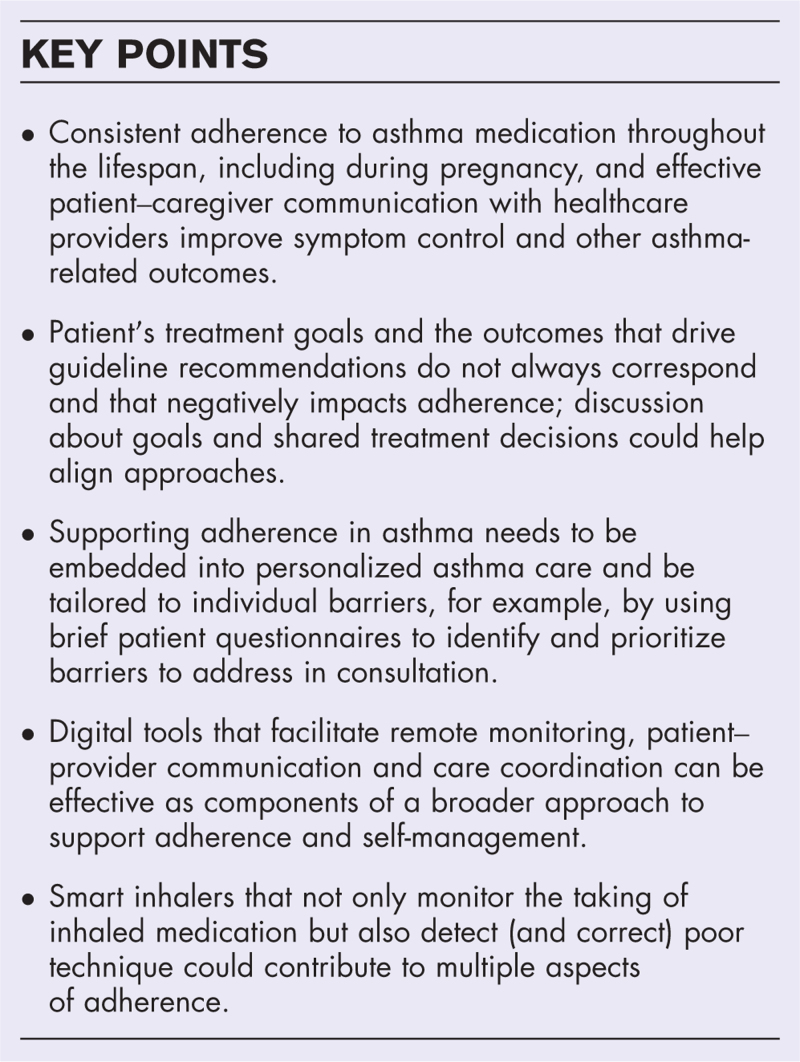
no caption available

## DOES ADHERENCE TO MEDICATION HAVE AN IMPACT ON CLINICAL OUTCOMES?

Recent studies reaffirm the established relationship between adherence and clinical outcomes such as poorer lung function [12], higher likelihood of oral cortico steroid (OCS) use [13] and short-acting beta-2 agonist (SABA) overuse [14^▪^], higher FeNO levels [15], and more exacerbations [12,14^▪^]. In addition, adherence to optimal inhaler technique may improve asthma-related outcomes [12].

A study [14^▪^] categorized patients included in the Swedish National Airway Register (SNAR) according to GINA treatment steps [16] and reported adherence data for steps 2–5. Adherence was worst for patients in step 2 (82%) and best for those in step 5 (62.2%), which is in line with research that suggests that severity of symptoms can be a predictor of adherence, whereas those with higher severity show better adherence [17]. This finding also reflects the ‘no symptom, no asthma’ phenomenon, which is a misconception that the absence of asthma symptoms means the condition requires no management; this underscores a disconnect between patient and HCP perceptions of asthma management [18]. Patients in step 5 had the highest proportion of exacerbations (33.8%) and exacerbations with hospitalization (25.3%). These findings highlight that, alongside nonadherence, factors such as comorbidities (diabetes, allergies, being an ex-smoker [13,14^▪^]) can contribute to uncontrolled asthma.

In a survey of Italian patients with mild-to-moderate asthma, reports of low adherence to inhaled corticosteroids (ICS) were common, resulting in OCS use to treat exacerbations. Patients expressed concerns about both ICS and OCS use and potential side effects [13]. Patients expressed being satisfied with their asthma therapy, in particular with the aspect of fast relief of symptoms. These findings emphasize the importance of open communication between patient and HCP to discuss any misalignments in the therapeutical approach [19,20].

The relationships between adherence and outcomes may manifest differently in specific patient sub-groups, requiring an individualized approach. Up to two-thirds of pregnant women with asthma discontinue asthma medications during pregnancy, putting them at increased risk for adverse outcomes, often resulting in asthma exacerbations that can trigger adverse maternal and neonatal outcomes [21]. For younger people with asthma, parents/caregiver adherence support and positive attitudes towards adherence are established predictors for adherence, asthma control, and other clinical outcomes [22]. In addition, parents/caregivers and young patients’ mental health can affect adherence, asthma care, and vice versa. Well controlled asthma, adherence to treatment, and a reduction in treatment were the primary predictors of positive mental health in young patients with asthma and their parents/caregivers [23^▪^]. These findings indicate the need for a patient and HCP relationship that is built on trust, especially with more vulnerable patient groups [24,25].

## WHAT FACTORS INFLUENCE ADHERENCE IN DIFFERENT PATIENT GROUPS?

Adherence support needs to be personalized to different demographic groups [9], and age seems to be relevant. Particularly, older adults (over 50) have better adherence than children and young people. In a study comparing adolescents (13–18 years), young adults (18 – 26 years), and adults (older than 26 years) regarding their adherence to ICS and ICS+LABA; adherence to ICS was similar between the age groups [26]. In contrast, adherence to ICS+LABA was best in adults older than 26 years. Older age (over 50), and diagnosis at older age (over 60) have been reported as predictors of higher adherence [17,27]. There is evidence that being from an ethnic minority background was a risk factor for nonadherence [28,29^▪^]. However, for young people, this effect disappeared when income and insurance status were controlled for [29^▪^], suggesting that resources such as having funds, and access to healthcare are more important for adherence than knowing a patient's ethnicity.

The way healthcare is delivered, and asthma is treated, is one of the most relevant factors for good adherence [30]. Poor adherence can be associated with receiving care in multiple sites [28]. For many patients, having care delivered on multiple sites can mean interactions with multiple HCPs and conflicting medication regimens, which can lead to misunderstandings and confusion about the roles of different medications [19] and negatively affect adherence. Similarly, patients and physicians can have different treatment goals. For patients, reducing exacerbation risk is the most important treatment goal, whereas, for physicians, it is asthma control [17]. This discrepancy can result in unfavorable treatment choices in patients, such as SABA overuse. However, some studies found that SABA overuse was associated with good ICS adherence [17,28]. In this case, this might be a sign of poorly controlled asthma [17]. Discrepancies exist between patient and HCP perceptions of what constitutes ‘good’ asthma control. Although patients have lived experience of their condition, they often lack the specialized knowledge required to effectively articulate their behaviors, symptoms, and lifestyle in ways that align with HCP asthma management [31], which in turn contributes to misunderstandings of medication regimen [19].

Recent findings suggest that patients with poor asthma control and adherence are more likely to report higher levels of anxiety and depressive symptoms [32^▪^,^33^]. Therefore, in clinical practice, it can be useful to understand if patients struggle with their mental health, because this can affect asthma self-management behaviors negatively. Particularly in young patients, ‘asthma burnout’ (feeling tired of having asthma) was identified as a barrier to adherence [29^▪^], which can be understood as ‘stigma’ and is widely recognized as a barrier to adherence, especially in children and young people [34].

Nonadherence to ICS is a global problem [35] and the studies that are part of this review reflect this to a certain degree. We were able to integrate studies from around the globe (e.g. Saudi-Arabia [33,36]; Turkey [27]; China [32^▪^]). The results on adherence determinants demonstrate that they are comparable across countries and therefore, the findings may be applicable in a range of clinical contexts.

## HOW CAN ADHERENCE BE SUPPORTED IN DIFFERENT CLINICAL SETTINGS?

Optimizing adherence is part of routine asthma care, but our review findings on the prevalence of suboptimal adherence and its many causes and consequences point to the necessity of pursuing continuous quality improvement and innovation. Table 1 shows adherence support interventions reported in the recent studies included in this review, illustrating the diversity of settings, target populations, and adherence support strategies at different stages of intervention development, evaluation, and implementation.

**Table 1:** Examples of recent studies on adherence support interventions in asthma

Article	Country	Setting	Population	Type of study	Intervention
Achterbosch *et al.*[37]	NL	Primary and secondary care	At least 18 years, asthma and/or COPD; using LABA, LAMA, and/or ICS for at least 3 months	Observational usability study	Test of Adherence to Inhalers (TAI) toolkit for assessment and selection of intervention on different adherence determinants, including a patient self-report questionnaire and recommendations to HCPs for adherence support; the kit was used in one consultation for selecting tailored interventions; intervention execution was not part of the study
Gao *et al.*[38]	China	Secondary care	5–12 years; uncontrolled bronchial asthma (GINA); initiating ICS/LABA	Randomized controlled trial	Asthma education, short-message (SMS) follow-up to parents before each dose for 12 weeks, telephone notice for follow-up visit (weeks 1, 4 and 12), medication use and inhaler technique assessed at each visit.
Halterman *et al.*[39]	US	Secondary care linking to primary care and school settings	3–12 years, persistent or poorly controlled asthma, presenting to the ED for asthma exacerbation	Randomized controlled trial	Telemedicine-enhanced Asthma Management through the Emergency Department (TEAM-ED) including school-based telemedicine follow-up (primary care), point-of-care prompting guideline-based care, opportunity for two additional telemedicine follow-ups
Mosnaim *et al.*[40]	US	Not specified	At least 13 years, uncontrolled asthma (Asthma Control Test score <19), prescribed ICS/LABA and SABA	Randomized controlled trial	Digihaler System used for 24 weeks, consisting of 2 Digihaler inhalers for maintenance and reliever medication, a patient App and web-based Dashboard used by clinicians to review inhaler use and support decision-making
O’Neill *et al.*[41]	Northern Ireland	Tertiary care	Adults accessing the asthma outpatient service at a local tertiary hospital	Service improvement project	Video-direct observation of therapy (v-DOT) app, instruction on correct inhaler use, and request to upload video recordings of each inhaler use over 6 weeks, reviewed by clinician daily for rating inhaler technique and option of patient contact for tailored feedback
Radu *et al.*[42]	US	School settings	6–18 years, persistent asthma (prescription of daily ICS) and poorly controlled asthma (Asthma Control Test score <19 or asthma exacerbation in previous year)	Qualitative evaluation of participant perspectives on intervention implementation	Remote Asthma Link (RAL) is a school-linked text-message intervention consisting of text messages for parents to administer medication and shared information with school nurse for follow-up and care coordination with pediatric care provider
Visser *et al.*[43]	NL	Primary care	At least 18 years, asthma and/or COPD; using LABA, LAMA and/or ICS dispensed in the last 4 months	2-arm nonrandomized controlled implementation trial	Respiratory Adherence Care Enhancer (RACE) questionnaire and two consultations (in-person or online; 5 weeks apart) to identify, visualize and address individual barriers to self-management

COPD, chronic obstructive pulmonary disease; ICS, inhaled corticosteroids; LABA, long-acting beta agonists; LAMA, long-acting muscarinic antagonists; NL, Netherlands; US, United States.

Conducting implementation research to adapt the intervention content and format to their target settings is essential for successful adoption in routine practice. Two interventions developed in the Netherlands for adults with asthma and/or COPD provide relevant examples.

(1) The Test of Adherence to Inhalers (TAI) toolkit [37] is based on research evidence of determinants and effective interventions for supporting inhaler adherence [44,45]. Determinants related to three types of nonadherence labeled as deliberate, unconscious, and sporadic were mapped onto intervention options pertaining to education and/or counseling, changes in medication plan, inhalation instruction, reminders, and strategies for coping and habit formation. The TAI toolkit prototype is a desktop helper in paper format and includes a patient self-report questionnaire and scoring instructions, and information for HCPs on the TAI items and practical tips for personalized intervention based on the TAI score. The toolkit was perceived as easy to use and adapt to current practice. It provided sufficient information for HCPs and could be supported by enhanced training within existing educational programs and integrated into national protocols and electronic records.
(2) The second intervention is based on a previously validated online questionnaire, the Respiratory Adherence Care Enhancer (RACE), that identifies self-management barriers, which are then presented to the patients as visual feedback to guide intervention choice [43]. In this implementation trial, the intervention was delivered by pharmacy students with brief training in patient-centered care and informed by evidence-based and theory-based intervention strategies. The RACE intervention was positively perceived by patients, who provided practical feedback on how this type of adherence support could be implemented in pharmacy care. No differences were observed between groups on disease control.

User feedback to both these interventions highlighted the potential of including more digital elements in the intervention packages.

A recent Cochrane review of digital interventions for optimizing adherence to asthma controller medication showed that digital tools are increasingly present in medication adherence support and respiratory care and likely to improve asthma control and quality of life and reduce exacerbations [46]. As a more recent example, an RCT conducted in the United States assessed the effectiveness of a digital inhaler system for improving asthma control in adolescents and adults with asthma. The Digihaler System consisted of two digital inhalers – for maintenance and reliever medication – linked to a patient app and web-based dashboard where HCPs could access usage data. Optimizing inhaler technique and adherence was the main causal mechanism targeted, via medication intake reminders, feedback on behavior, and stimulating interaction with HCPs – likely to represent opportunities for further targeted intervention on individual adherence determinants. Another digital tool tested for supporting monitoring and supervising asthma treatment in adults is the video-direct observation of therapy (v-DOT), an app for video recording inhaler use and receiving daily assessment and tailored feedback from the treating clinician for adjusting inhaler technique if required. A service development project in Northern Ireland evaluated the use of v-DOT in 10 patients and their clinicians, and showed high engagement, usability, and satisfaction with the tool over 7 weeks, although five of them requested support with technical issues during this time. However, implementing such digital tools in clinical settings requires training of both patients and HCPs on technical aspects and relies on HCP time and behavioral intervention skills for addressing individual barriers identified digitally or during consultation. Therefore, it is important to carefully consider the patient groups for which these interventions can be implemented, as well as implementation costs.

Optimizing adherence in children with asthma tends to focus primarily on parents’ behaviors and support them via education and reminders for habit formation. Some interventions in secondary care are initiated with the prescription of new medication and/or following an acute episode of asthma exacerbation. An intervention developed and tested in China [38] included several follow-up actions (SMS, telephone, in-person visits) to support habit formation and monitor and give feedback on inhaler technique and adherence. These additional actions were shown to lead to better asthma control after 12 weeks compared to usual care. The Telemedicine-enhanced Asthma Management through the Emergency Department (TEAM-ED) intervention [39] developed in the United States also involved parents/caregivers, as well as school-based clinical telehealth assistants, and the child's primary care provider who were trained to deliver specific preventive asthma care activities including education provision and development of a treatment plan. TEAM-ED improved follow-up and preventive care after emergency department visit, though failed to improve medication adherence and reduce asthma morbidity at 12 months, suggesting that more sustained support is necessary for improving outcomes. The Remote Asthma Link (RAL) intervention is a similar intervention involving parents of children with asthma, their school nurses and their pediatric care providers and consisting of daily text messages to parents to prompt medication administration, and information shared with school nurses who perform weekly remote check-ins to promote adherence and communicate with pediatric providers for care coordination [42]. The RAL intervention, previously found feasible and effective in improving medication adherence and reducing asthma exacerbations in the COVID-19 context [47], was positively perceived by participants, who also identified several barriers and solutions for its implementation, and possibilities for expansion to other target groups.

## IMPLICATIONS FOR CLINICAL PRACTICE

**FIGURE 1:**
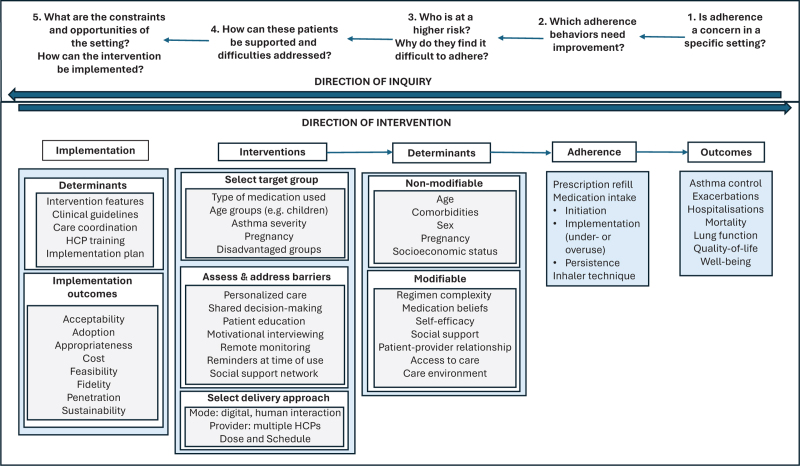
Conceptual approach to optimizing adherence to medication in asthma.

Research continues to indicate a high prevalence of suboptimal adherence in asthma, with significant impact on clinical outcomes and a complex network of determinant factors. It also describes promising approaches to improve adherence by a personalized approach to patient support and using digital tools to facilitate monitoring, communication, and coordination. How could this new evidence inform quality improvement work in specific clinical settings? Figure 1 summarizes a conceptual approach to interventions that can guide research on, and adoption of, adherence support innovation in clinical practice. Informed by established theory and methodological recommendations [48–50], it proposes five questions for reflection on optimizing adherence in a specific setting.

(1) First, new efforts dedicated to improving adherence need to be informed by an assessment of the current situation and its impact on clinical outcomes: is adherence a concern in this setting? The studies we reviewed show that this is the case in many settings globally. Confirming this hypothesis locally and ascertaining the extent of the problem gives compelling arguments for prioritization and underpins involvement of key stakeholders.
(2) Second, it is important to clarify which adherence behaviors are particularly challenging for patients and HCPs and need improvement? There is still a complex dynamic between underuse of ICS, poor inhaler technique, and overuse of SABA and OCS, resulting from a multifaceted interaction between prescriber, patient, and caregiver behaviors. Understanding this interplay and sequence of behaviors is essential for effective support. Given the variability of asthma, patient empowerment would require supporting them to adjust their preventer medication according to their asthma action plan [51]
(3) Third, the behavioral determinants with the highest relevance in this setting need to be identified and prioritized to understand who is most at risk, and why do they struggle with adherence? Among the diverse range of determinants reported in the literature, a key distinction is to be made between nonmodifiable factors (such as age, sex, socioeconomic status) that may inform the selection of patient groups in need of more support, and modifiable factors (such as regimen complexity or medication beliefs), which may be the focus of the intervention itself.
(4) Once setting-specific information on adherence outcomes, behaviors, and determinants is obtained, a fourth question can be answered on how patients can be supported, by focusing intervention on specific target groups and selecting strategies to address the modifiable factors identified. The interventions reviewed above illustrate different options, such as supporting a broad group of inhaler users by a personalized approach that prioritizes intervention components (TAI and RACE) [37,43] or focusing on enhancing support in school settings for children with asthma (TEAM-ED, RAL) [39,42].
(5) A fifth question is increasingly asked in adherence research and has proven crucial for clinical practice: how can interventions be implemented? Understanding the constraints and opportunities in the setting in which an intervention is intended to function provides key information for adjusting the intervention content and delivery approach, which is also evaluated on implementation outcomes, such as feasibility or acceptability [37,41–43].

Following this sequence of five questions from adherence outcomes to implementation considerations enables a causal, systematic approach to optimizing adherence in new clinical settings.

## CONCLUSION

Adherence to medication continues to be identified as a barrier for improving clinical outcomes in different patient groups globally. Poor inhaler technique, underuse of ICS, and overuse of SABA and OCS are still highly prevalent despite strong recommendations in clinical guidelines for sustained assessment and support of appropriate medication use. Different adherence determinants and intervention strategies may be relevant for different patient groups. By following a systematic approach to understanding adherence behaviors in context, research evidence can inform continued improvement of adherence support as part of personalized asthma care.

## Acknowledgements

*None.*

### Financial support and sponsorship

*None.*

### Conflicts of interest

*H.P. declares multiple research grants related to delivery of asthma care, implementation of supported self-management, and digital respiratory healthcare. She has received speaker fees from Teva and Sandoz Pharmaceuticals for nonpromotional talks in sponsored symposia.*

## REFERENCES AND RECOMMENDED READING

Papers of particular interest, published within the annual period of review, have been highlighted as:

▪ of special interest
▪▪ of outstanding interest
